# ADVERSE CHILDHOOD EXPERIENCES AS A PREDICTOR OF HEALTH BEHAVIORS IN WOMEN

**DOI:** 10.1093/geroni/igad104.0353

**Published:** 2023-12-21

**Authors:** Karina Tavares, Cindy Tsotsoros, Skye Leedahl, Jessica Tsotsoros

**Affiliations:** University of Rhode Island, Kingston, Rhode Island, United States; University of Rhode Island, Kingston, Rhode Island, United States; University of Rhode Island, Kingston, Rhode Island, United States; University of Oklahoma Health Sciences Center, Tulsa, Oklahoma, United States

## Abstract

Individuals who experience adversity during childhood are at a higher risk of negative health outcomes in adulthood. This study aims to identify whether adverse childhood experiences (ACEs) influence health lifestyles throughout adulthood. This was accomplished by collecting data from 233 women aged 18-25 and 65-85 who reported either no ACEs or three or more ACEs. Demographic indicators, the 10-item ACEs questionnaire (Felitti et al., 1998), and the Health Promoting Lifestyle Profile (HPLP-II; Walker, Sechrist, & Pender, 1987) were examined. ACEs were categorized into three variables: abuse, neglect, and household dysfunction. The HPLP-II was categorized by its six subscales: health responsibility, nutrition, spiritual growth, physical activity, stress management, and interpersonal relations. When comparing the ACEs and no ACEs groups, t-tests revealed significantly different scores for overall HPLP-II and the six subcategories showing that individuals without ACEs have higher scores on health behaviors. A structural equation model was calculated using the three ACE categories and six health domains. Substantial differences were observed in the variance captured for each of the six health behavior measures. Findings indicate that abuse significantly predicts physical activity, stress management, and spiritual growth (β=-.21, -.23, -.20); neglect significantly predicts interpersonal relationships and spiritual growth (β=-.17, -.18); and household dysfunction significantly predicts health responsibility, nutrition, stress management, and interpersonal relations (β=-.20, -.22, -.10, -.17). The present investigation extends research in displaying that ACEs play a significant role in future health behaviors, with household dysfunction being the greatest predictor.

